# Integrated Genomic Analysis of the 8q24 Amplification in Endometrial Cancers Identifies *ATAD2* as Essential to *MYC-*Dependent Cancers

**DOI:** 10.1371/journal.pone.0054873

**Published:** 2013-02-05

**Authors:** Maria B. Raeder, Even Birkeland, Jone Trovik, Camilla Krakstad, Shyemaa Shehata, Steven Schumacher, Travis I. Zack, Antje Krohn, Henrica MJ. Werner, Susan E. Moody, Elisabeth Wik, Ingunn M. Stefansson, Frederik Holst, Anne M. Oyan, Pablo Tamayo, Jill P. Mesirov, Karl H. Kalland, Lars A. Akslen, Ronald Simon, Rameen Beroukhim, Helga B. Salvesen

**Affiliations:** 1 Department of Obstetrics and Gynecology, Haukeland University Hospital, Bergen, Norway; 2 Department of Clinical Medicine, University of Bergen, Bergen, Norway; 3 Department of Cancer Biology and Department of Medical Oncology, Dana-Farber Cancer Institute, Boston, Massachusetts, United States of America; 4 The Broad Institute, Cambridge, Massachusetts, United States of America; 5 Department of Biophysics, Harvard University, Boston, Massachusetts, United States of America; 6 Department of Pathology, University Medical Center Hamburg-Eppendorf, Hamburg-Eppendorf, Germany; 7 The Gade Institute, University of Bergen, Bergen, Norway; 8 Department of Pathology, Haukeland University Hospital, Bergen, Norway; 9 Department of Microbiology and Immunology, Haukeland University Hospital, Bergen, Norway; 10 Departments of Medicine, Harvard Medical School and Brigham and Women’s Hospital, Boston, Massachusetts, United States of America; Peking University Health Science Center, China

## Abstract

Chromosome 8q24 is the most commonly amplified region across multiple cancer types, and the typical length of the amplification suggests that it may target additional genes to *MYC*. To explore the roles of the genes most frequently included in 8q24 amplifications, we analyzed the relation between copy number alterations and gene expression in three sets of endometrial cancers (N = 252); and in glioblastoma, ovarian, and breast cancers profiled by TCGA. Among the genes neighbouring *MYC*, expression of the bromodomain-containing gene *ATAD2* was the most associated with amplification. Bromodomain-containing genes have been implicated as mediators of MYC transcriptional function, and indeed *ATAD2* expression was more closely associated with expression of genes known to be upregulated by MYC than was *MYC* itself. Amplifications of 8q24, expression of genes downstream from MYC, and overexpression of *ATAD2* predicted poor outcome and increased from primary to metastatic lesions. Knockdown of *ATAD2* and *MYC* in seven endometrial and 21 breast cancer cell lines demonstrated that cell lines that were dependent on MYC also depended upon ATAD2. These same cell lines were also the most sensitive to the histone deacetylase (HDAC) inhibitor Trichostatin-A, consistent with prior studies identifying bromodomain-containing proteins as targets of inhibition by HDAC inhibitors. Our data indicate high ATAD2 expression is a marker of aggressive endometrial cancers, and suggest specific inhibitors of ATAD2 may have therapeutic utility in these and other MYC-dependent cancers.

## Introduction

Endometrial carcinoma is the most common pelvic gynecologic malignancy, with a lifetime risk among women of 2–3% [1]. Approximately 75% of tumors are confined to the uterine corpus at diagnosis and are resected. However, 15%–20% of these tumors relapse. These tumors, and tumors that are metastatic at presentation, respond poorly to chemotherapy or radiation and are generally fatal [1], [2].

There is a need for novel markers to identify patients with high risk of relapse, and to develop new therapies for patients with metastatic disease [3], [4]. Unfortunately, research towards these goals is heavily underrepresented in endometrial cancer compared to other cancer types such as breast and ovarian cancers. One approach is to identify genes that, when altered by somatic genetic events, drive tumor progression. These alterations can then serve as markers of aggressive cancers and the genes can serve as potential therapeutic targets.

The most frequent focal amplification in endometrial cancer is on 8q24 [5]. Indeed, 8q24 is the most commonly amplified region across multiple cancer types [6], and this amplification is a negative prognostic marker in several cancers [7]. Although *MYC* is a likely target [6], the effects of this amplification in endometrial cancer have never been dissected. Indeed, it is possible that it targets multiple genes, as has been shown for amplifications elsewhere in the cancer genome [8]. For example, a neighboring gene, *ATAD2*, has been found to be a co-regulator of *MYC* and overexpression of *ATAD2* has been associated with poor prognosis in breast, lung, and prostate cancers [9], [10], [11].

We explore the role of the 8q24 amplification in endometrial cancer through integrative genomic analyses of primary and metastatic endometrial cancers with comprehensive clinical data, and identify *ATAD2* as an additional target of the 8q24 amplification in these cancers. We identify copy number gain of *ATAD2* as a regulator of *ATAD2* expression, present the first data linking *ATAD2* overexpression to *MYC* activation, and provide functional data suggesting ATAD2 as a therapeutic target in MYC-dependent cancers.

## Materials and Methods

### Ethics Statement

The collection of endometrial carcinoma primaries and metastases for this study was approved by the Norwegian Data Inspectorate (961478-2), Norwegian Social Sciences Data Services (15501) and the “Regional Research Ethics.

Committee in Medicine, Western Norway” (reference 052.01). All the participants gave written informed consent.

### Patient Series

Endometrial carcinoma primaries and metastases were collected from patients treated at Haukeland University Hospital, Norway as previously described [5]. Tumors collected for the primary investigation and qPCR validation series were frozen immediately upon resection; tumors collected for FISH were formalin fixed and paraffin embedded. Patients were followed from primary surgery until October 2010 or death. The copy-number profiles of the primary investigation series, and the expression profiles (Agilent 21 k and 22 k oligoarrays) from a subset of 57 tumors, were published previously [5].

### RNA Analysis

RNA was extracted and hybridized to Agilent 44K arrays (Cat.no. G4112F) according to manufacturer’s instructions and as previously described [5]. Signal intensities were evaluated using BRB-ArrayTools (National Cancer Institute, USA). The arrays were batch median normalized.

### Real-time Quantitative PCR

cDNA was synthesized from 1 µg RNA using High capacity RNA to cDNA kits (Applied Biosystems). Expression of *ATAD2* and *MYC* was determined using TaqMan gene expression assays Hs00204205 and Hs00905030 respectively (Applied Biosystems) and all samples were run on microfluidic cards per manufacturer’s instructuions, using GAPDH-Hs99999905_m1 as endogenous control. Samples were run in triplicate and analyzed in RQ manager (Applied Biosystems).

### FISH

Tissue microarrays (TMAs) representing the highest-grade areas in each tumor were prepared as previously reported [12] and treated at 56C° overnight before hybridization. FISH was done using the MYC Spectrum Orange FISH probe kit and Chromosome enumeration probe 8 (CEP8) (Vysis) according to manufacturer’s instructions, as previously reported [13]. Counting was performed in areas of optimal tissue digestion and no overlapping nuclei. Probe and control signals were counted in 40–60 cells within areas of optimal tissue digestion and no overlapping nuclei. Amplifications were scored when the MYC/CEP8 ratio was >1.0.

### TCGA Validation Dataset

We accessed level 3 data from the TCGA data portal in November and December 2010. For breast cancer we obtained gene expression data for 279 tumors and 24 normal controls (Agilent 244K expression arrays), and copy-number from 176 tumors (Affymetrix SNP 6.0 Arrays). For ovarian cancer we obtained gene expression (Agilent 244K expression arrays) and copy-number (Agilent 1M arrays) data from 514 and 489 tumors, respectively. For glioblastoma we obtained gene expression data from 385 tumors and 10 normal controls (Affymetrix U133A arrays) and copy-number data from 261 tumors (Agilent 244K arrays).

### Cell Viability

Lentiviral vectors encoding shRNAs specific for *ATAD2*, *MYC*, and the controls *GFP*, *LACZ1*, and *LACZ2* (Table S1) were obtained from The RNAi Consortium. Lentivirus was produced by transfection of 293T cells with vectors encoding each shRNA (5 µg) with packaging plasmids encoding PSPAX2 and PDM2.G using Fugene HD (Roche). Lentivirus-containing supernatant was collected 48 and 72 h after transfection, pooled, and stored at −80 °C. Cells were infected in polybrene-containing media, centrifuged at 1,000 g for 30 min, and selected in puromycin (2.5 µg ml^−1^) starting 24 h after infection.

Cancer cell lines were obtained from ATCC, DSMZ, ECACC and HSRRB, and grown according to supplier’s instructions (Table S2). Cell viability after RNAi was measured in 96-well plates. Eight wells seeded with cells were infected using 1∶30 dilutions of virus containing each shRNA. Half of the wells underwent puromycin selection, and cell viability was measured using Cell-Titer Glo (Promega) one week later. The values from each quadruplicate were averaged; “outlier” wells were excluded if the replicate wells had SD/mean >0.2 and excluding the well improved the variance. The mean ATAD2- and MYC- hairpin values were normalized to the mean values from the GFP control.

To determine Trichostatin-A sensitivity, Trichostatin-A (Sigma) (0.040 to 10 µM) and vehicle (DMSO) control were each added to three wells containing each cell line on 96-well plates. Cell viability was determined after 72 hours using Cell-Titer Glo (Promega).

### Immunoblotting

Cells were washed with PBS, harvested, lysed using RIPA lysis buffer with protease and phosphatase inhibitors, and centrifuged at 16,000×g. Supernatant was mixed with 4X SDS sample buffer, boiled for 7 minutes, and subjected to SDS-PAGE on 4–12% gradient gels. Blots were probed with antibodies against ATAD2 (HPA029424, Sigma), MYC (sc-764, Santa Cruz) and actin (sc-1615, Santa Cruz).

### Statistics

Molecular data was related to clinical phenotype using Pearson’s χ^2^ or two-sided Student’s t test as appropriate. We used multivariate linear regression analysis for the prediction of *ATAD2* expression levels. Univariate and multivariate survival analyses were performed by log rank and Mantel-Cox methods, respectively. “MYC signaling strength” and gene expression levels were presented as Z-scores.

## Results

### Assessment of *MYC* as a Target of the 8q24 Amplification

Extensive biological data support *MYC* as an oncogene [14], and 8q24, harboring *MYC*, is the most common amplified region across multiple cancer types [6]. However, the importance of MYC activation in endometrial cancer is essentially unknown.

We performed an integrated analysis of copy-number and expression data to look for evidence that *MYC* is a target of 8q24 amplification in endometrial cancer. We evaluated expression data from a series of 82 endometrial cancers obtained in a single county in Norway, with corresponding genome-wide copy-number data from 70 tumors (the “primary investigation series”). Sixteen of these tumors (23%) had 8q24 amplification. Most of these amplifications were low-level, ranging up to a copy-number of 4.7. We validated our results in four additional datasets. Two of these represent samples with genome-wide expression profiling: an “internal validation series” for which we generated data from 40 primary and 19 metastatic endometrial cancers recruited from the same region in Norway, and an “external validation series” representing previously published expression profiles from 111 tumors [5]. The other two validation sets represent samples analyzed with focused assays: a “qPCR series” of 162 samples and a “FISH series” of 399 samples. Patient characteristics and histopathological variables for all of our internal datasets are shown in Table S3.

**Figure 1:**
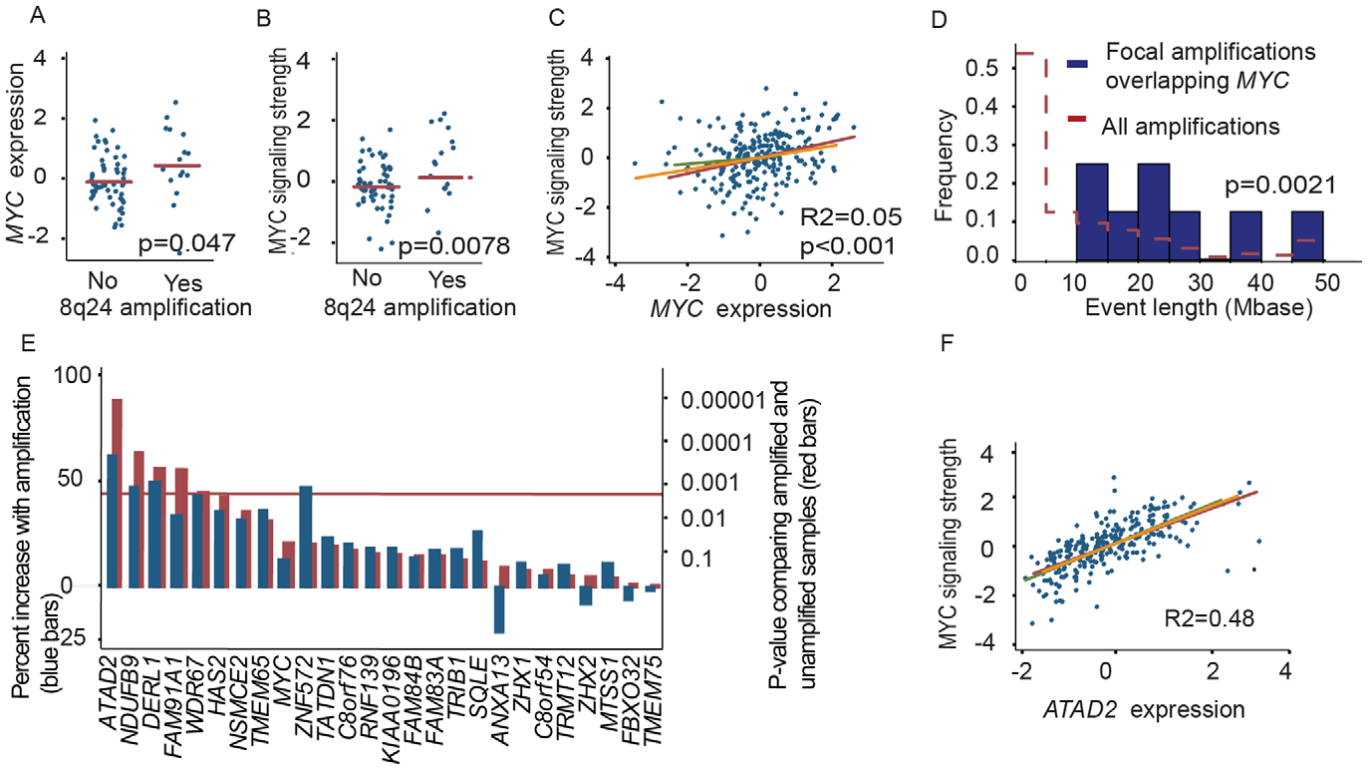
*MYC, ATAD2* and 8q24 associations. (a) *MYC* expression and (b) MYC signaling are both increased among endometrial cancers with 8q24 amplification. (c) Variations in *MYC* expression only explain a small proportion of the variation in MYC signaling. Linear fits are shown in red, yellow, and green for the primary investigation series, internal validation series, and external validation series, respectively. (d) The lengths of the amplifications that contain *MYC* are significantly larger than expected compared to amplifications observed elsewhere in these cancers. (e) Among 26 genes in the 8q24 peak with corresponding expression data, expression of *ATAD2* is most strongly and significantly associated with amplification. Blue bars show the percent increase in gene expression and red bars show the p-values. The significance threshold is Bonferroni-corrected for multiple hypotheses. (f) Expression of *ATAD2* is highly correlated with MYC signaling strength. Linear fits are shown as in panel c.

We found that both *MYC* and genes upregulated by MYC were overexpressed in endometrial cancers with 8q24 amplification relative to endometrial cancers without it (p = 0.047 and p = 0.0078, respectively) (Figures 1a–b). We used a previously published list of 68 genes found to be upregulated by MYC across multiple contexts and assays (Table S4; www.myccancergene.org) [15] and scored their overexpression (“MYC signaling strength”) using GSEA [16]. We also tested five additional MYC activation signatures obtained from the “Gene Set Enrichment Database”, reflecting the activation of MYC in different contexts. Four of these were expressed at higher levels in endometrial cancers with 8q24 amplification (Figure S1a).

However, variations in *MYC* expression itself only explained a small proportion of variations in MYC signaling strength (R2 = 0.11, p = 0.002) (Figure S1b). We obtained similarly weak results in the internal and external validation datasets (R2 = 0.00, p = 0.34 and R2 = 0.06, p = 0.012, respectively; Figure S1b). Across all three datasets, variations in MYC expression only account for 5% of variations in MYC signaling strength (R2 = 0.05, Figure 1c).

Moreover, 8q24 amplifications are longer than the typical distribution of amplification sizes in endometrial cancer (p = 0.0021) (Figure 1c), and usually involve multiple genes. We therefore hypothesized that 8q24 amplifications may target additional genes, some of which may function through increasing MYC signaling. To identify these, we evaluated all 26 genes in the peak region of amplification on 8q24 for which we had expression data, to identify genes whose expression correlated most strongly with amplification.

### Expression of *ATAD2* Correlates Strongly with 8q24 Amplification and MYC Signaling

Expression of *ATAD2* was more strongly associated with amplification of 8q24 than was expression of any other gene in the peak region of the amplification (p-value = 2.77E-06) (Figure 1d). Four other genes, *NDUFB9*, *DERL1*, *FAM91A1*, and *WDR67,* were significantly upregulated by 8q24 amplification, though less strongly than *ATAD2*.

Expression of *ATAD2* also correlated with MYC signaling strength more strongly than did expression of any other gene in the 8q24 peak region (R2 = 0.48, p<0.001; Figure 1e, Figure S1b). Indeed, the association between *ATAD2* expression and MYC signaling strength was observed even among samples without 8q24 amplification (R2 = 0.48, p<0.001). The correlation between *ATAD2* expression and MYC signaling strength was more than twice as strong as the next most significantly associated gene (*NDUFB9*) and stronger than for *MYC* itself (R2 = 0.05; Figure 1c). *ATAD2* is not one of the 68 genes in the MYC activation signature, and to our knowledge *MYC* has not been found to modulate expression of *ATAD2* [15]. However, ATAD2 was previously found to bind to MYC and to the E-box region of several MYC target genes, and ATAD2 levels were found to be limiting for MYC-dependent transcription [9].

Both genome-wide validation series also exhibited the correlation between MYC signaling and *ATAD2* expression (R2 = 0.54, p<0.001 and R2 = 0.45, p<0.001 in the internal and external validation series, respectively) and the relative lack of correlation with *MYC* expression (R2 = 0.00, p = 0.33 and R2 = 0.06, p = 0.012; Figures 1f and S1b). Expression of *ATAD2* also correlated with four of the five additional signatures of MYC activation, and correlated more strongly with these signatures than did expression of *MYC* itself (Table 1). The last signature showed no association with *ATAD2* or *MYC* expression.

**Table 1:** Associations between other MYC activation gene sets and *ATAD2*- and *MYC* expression.

		*ATAD2*				*MYC*			
Gene set		R2	P-value	P-value*		R2	P-value		P-value*
Schumacher myc up†		0.45	<0.001		<0.001	0.11		<0.001	0.001
Primary Investigation Series		0.43	<0.001		<0.001	0.16		<0.001	0.025
Internal validation Series		0.38	<0.001		<0.001	0.05		0.800	0.252
External validation Series		0.5	<0.001		<0.001	0.49		<0.001	0.026
Coller myc up‡		0.35	<0.001		<0.001	0.10		<0.001	0.002
Primary Investigation Series		0.36	<0.001		<0.001	0.19		<0.001	0.006
Internal validation Series		0.22	<0.001		0.001	0.03		0.186	0.427
External validation Series		0.43	<0.001		<0.001	0.10		0.001	0.08
Yu cmyc up§		0.62	<0.001		<0.001	0.05		<0.001	0.987
Primary Investigation Series		0.62	<0.001		<0.001	0.06		0.290	0.612
Internal validation Series		0.68	<0.001		<0.001	0.02		0.252	0.973
External validation Series		0.60	<0.001		<0.001	0.07		0.006	0.632
Myc oncogenic signature¶		0.20	<0.001		<0.001	0.23		<0.001	<0.001
Primary Investigation Series		0.27	<0.001		<0.001	0.19		<0.001	0.004
Internal validation Series		0.08	0.042		<0.001	0.32		<0.001	0.133
External validation Series		0.24	<0.001		<0.001	0.22		<0.001	<0.001
Lee myc up||		0.05	<0.001		0.001	0.02		0.150	0.147
Primary Investigation Series		0.02	0.175		0.175	0.00		0.825	0.79
Internal validation Series		0.00	0.656		0.587	0.00		0.612	0.553
External validation Series		0.18	<0.001		<0.001	0.18		0.002	0.051

R2 and p-values are derived from a linear regression of the sum of expression values within the gene set against *ATAD2* or *MYC* expression.

* Adjusted for *ATAD2* or *MYC* expression.

† Genes up-regulated in P493-6 cells (Burkitt’s lymphoma) induced to express MYC (Schumacher).

‡ Genes regulated by forced expression of MYC in 293T (transformed fetal renal cell).

§ Genes up-regulated in B cell lymphoma tumors expressing an activated form of MYC.

¶ Genes selected in supervised analyses to discriminate cells expressing c-Myc from control cells expressing GFP. Myc oncogeneic.

|| Genes up-regulated in hepatocellular carcinoma (HCC) induced by overexpression of MYC.

### Amplification of 8q24 and Expression of *ATAD2*, but not MYC, are Associated with Disease Progression

Among the 70 endometrial cancers for which we had genome-wide SNP array data, 8q24 amplification was associated with reduced progression-free survival (p = 0.024) and increased risk for disease-specific death (p = 0.043). Amplification of 8q24 was most frequent in non-endometrioid (p = 2.98E-05) and high-grade tumors (p = 2.90E-08) (Table S5), features also associated with aggressive cancers [17].

**Figure 2:**
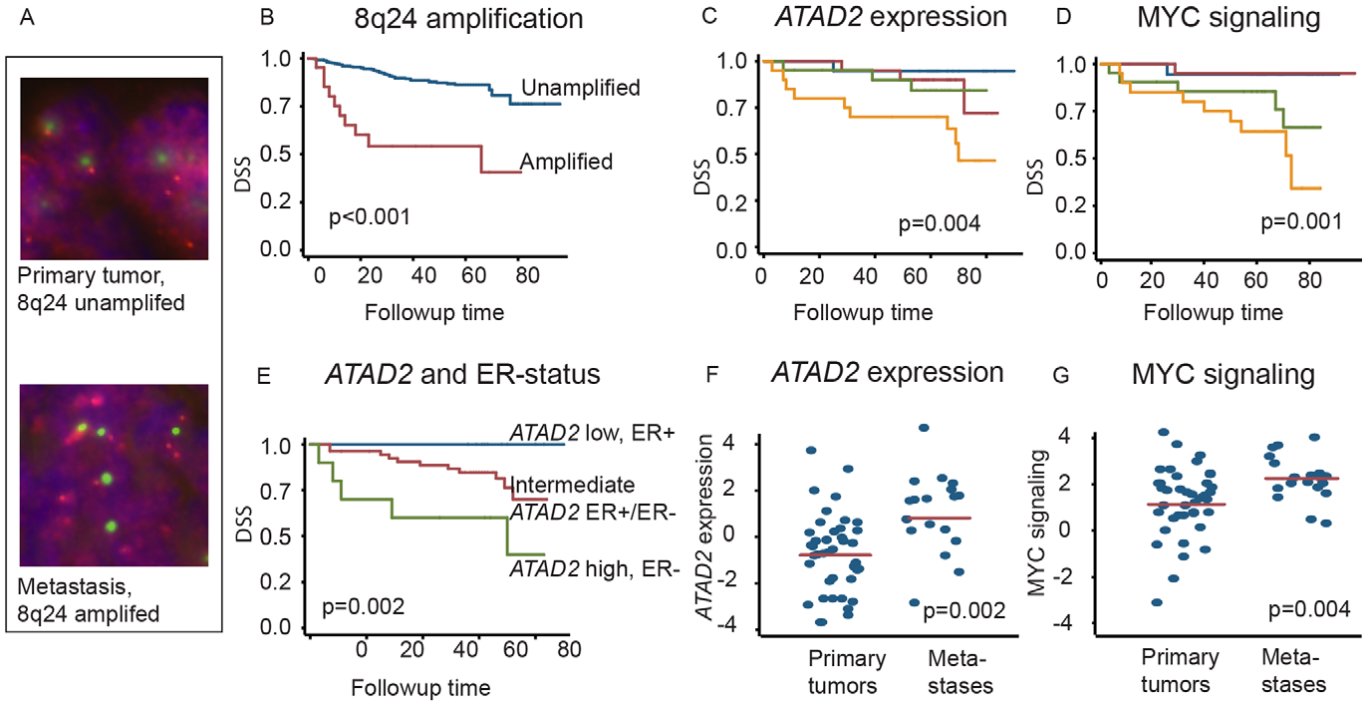
Amplification of 8q24, *ATAD2* overexpression and increased *MYC* signaling are associated with poor prognosis. FISH probes against 8q24 (red) and the chromosome 8 centromere (green) in a primary tumor and the paired metastasis show amplification only in the latter (a) (b) Among 399 patients assessed by FISH, those with 8q24 amplifications have worse outcome. In the primary investigation series, tumors among the highest quartiles of (c) *ATAD2* expression and (d) MYC signaling strength also had increased risk of disease-specific death. (e) Estrogen receptor negative (ER−) tumors with *ATAD2* expression in the top quartile were also associated with a high risk of disease-specific death; the risk was much lower among estrogen receptor positive (ER+) tumors with *ATAD2* expression in the bottom quartile. (f) *ATAD2* expression and (g) MYC signaling are both higher among metastases than primary tumors in the internal validation series.

We confirmed 8q24 amplification is associated with poor prognosis using FISH in an independent series of 399 endometrial cancers. Twenty cancers (5%) exhibited increased 8q24 copy-numbers relative to the chromosome 8 centromere (Figure 2a). These were associated with 64% 5-year survival, vs. 85% for cancers without 8q24 amplification (p<0.001) (Figure 2b). A similar pattern was seen for recurrence free survival (p = 0.001). Amplification of 8q24 was also associated with high FIGO stage (p = 0.003), non-endometrioid histological subtype (p<0.001), and high grade (p<0.001) (Table S5).

High expression of *ATAD2* and MYC signaling were also associated with increased risk of cancer progression (p = 0.003 and p = 0.015, respectively), cancer-specific death (p = 0.004 and p = 0.001) (Figures 2c–d), and other poor-prognosis features. *ATAD2* expression was higher in non-endometrioid, high-grade and ER negative tumors (p<0.001, p<0.001, and p = 0.02 respectively; Table S6); high MYC signaling was associated with poorly differentiated (p = 0.0016) and non-endometrioid (p<0.001) cancers. Expression of *ATAD2* was also negatively associated with expression of *ESR1* (R2 = 0.10, p = 0.005). Similarly, prior studies have found that *ATAD2* expression is higher in triple negative breast cancer tumors [18] and is downregulated by estrogen in cell culture [19].

Indeed, *ATAD2* expression was an independent predictor for disease-specific death (HR = 1.83, p = 0.027) and disease progression (HR = 1.62, p = 0.011) after adjusting for ER status. ER-negative tumors with upper-quartile *ATAD2* expression were particularly lethal (HR = 4.1, p = 0.002; Figure 2e).

We confirmed these results by assessing *ATAD2* expression by quantitative PCR and ER status by immunohistochemistry in our qPCR validation series of 162 additional tumors. Among these, ER-negative tumors with upper-quartile *ATAD2* expression were associated with even worse outcomes than in the primary series (HR = 6.8, p<0.001) (Figure S1c). High *ATAD2* expression also remained associated with increased risk of disease-specific death (p = 0.0043) and shorter progression-free survival (p = 0.016). After adjusting for ER status, *ATAD2* expression continued to predict disease-specific death (HR = 1.86, p = 0.018) but only trended towards an association with disease progression (HR = 1.31, p = 0.11). Expression of *ATAD2* was also higher in high-grade (p<0.001), non-endometrioid (p = 0.005), and ER-negative tumors (p = 0.02) (Table S6).

In contrast, *MYC* expression was not associated with progression or risk of disease-specific death in either our primary investigation series (p = 0.07 and p = 0.68 respectively) or the qPCR validation series (p = 0.53 and p = 0.28 respectively). High expression of *MYC* was associated with high grade in both series (p<0.001 and p = 0.02, respectively), and with non-endometrioid histology in the primary investigation series (p = 0.03) (Table S6).

Metastases also exhibit more 8q24 amplification, *ATAD2* expression, and *MYC* signaling strength than primary tumors. Relative to primary tumors, metastases exhibited 2.4× higher rates of focal 8q24 amplification by FISH (14.3%; p<0.007). Among the 399 patients in the FISH series, 49 had paired primary and metastatic tumors. Five of these samples (10%) did not exhibit 8q24 amplification in the primary but acquired it in the metastasis. Only one sample (2%) exhibited the opposite pattern. To examine *ATAD2* expression and MYC signaling, we also compared the 42 primary tumors with the 19 metastases in our internal validation series. Both *ATAD2* expression and MYC signaling strength were higher in the metastases (p = 0.002 and 0.004 respectively; Figure 2f–g), including among 8 patients with paired primary tumors and metastases (p = 0.01 and 0.05 respectively).

### Extension to Other Cancer Types and Normal Tissue

We also investigated whether 8q24 amplification is associated with increased *ATAD2* expression in other cancer types. Specifically, we used data from 514 ovarian cancers, 279 breast cancers, and 385 glioblastomas from The Cancer Genome Atlas [20], [21]. These included expression data from adjacent normal tissue for 24 breast cancers and 10 glioblastomas. 8q24 amplifications were observed in 72% (N = 126) of the breast cancers, 74% (N = 364) of the ovarian cancers, and 10% (N = 27) of the glioblastomas. *ATAD2* was co-amplified to the same level as *MYC* in nearly all tumors (as it was among our endometrial cancers; Figure S1d–g).

Expression of *ATAD2* correlated with 8q24 amplification among all three cancer types (R2 = 0.47, 0.11, and 0.36 for breast cancers, glioblastomas, and ovarian cancers respectively; p<0.001 in all cases; Table S7), and was 2.6× and 2.5× higher among the cancers relative to normal tissue in breast cancer and glioblastoma, respectively (p<0.001 in both cases). *MYC* expression correlated less strongly with 8q24 amplification in all three cancer types (R2 = 0.12, 0.07, and 0.10 for breast cancers, glioblastomas, and ovarian cancers respectively; p<0.001 in all cases). *MYC* expression in breast cancers was surprisingly half that of normal tissue (p<0.001); in glioblastoma it was higher by a factor of 3.5 (p<0.001).

*ATAD2* expression also correlated with MYC signaling in all three cancer types (R2 = 0.11, 0.21, and R2 = 0.31, respectively for breast cancers, glioblastomas, and ovarian cancers; p<0.001 in all cases), and was more strongly correlated with MYC signaling strength than *MYC* expression was (R2 = 0.10, p<0.001; R2 = 0.09, p<0.001; and R2 = 0.02, p = 0.003 in the three cancer types).

### *ATAD2* Expression is Correlated to *E2F* Gene Expression and *ATAD2* Copy Number in an Additive Manner

We also explored the relative contributions of E2F, estrogen, and copy-number on ATAD2 expression. The *ATAD2* promoter region contains binding sites for several E2F proteins and previous functional data have shown that E2F increases *ATAD2* expression in cell culture [9], [22]; *ATAD2* has also been induced by estrogen [23]. In our data, expression of every *E2F* transcription factor was associated with *ATAD2* expression, but only the inclusion of *E2F1*, *E2F2* and *E2F8* improved the overall fit of a model predicting *ATAD2* expression from *ATAD2* copy-number and *ESR1* expression. The expression levels of these three genes were highly correlated, and we focused on *E2F1*.

**Figure 3:**
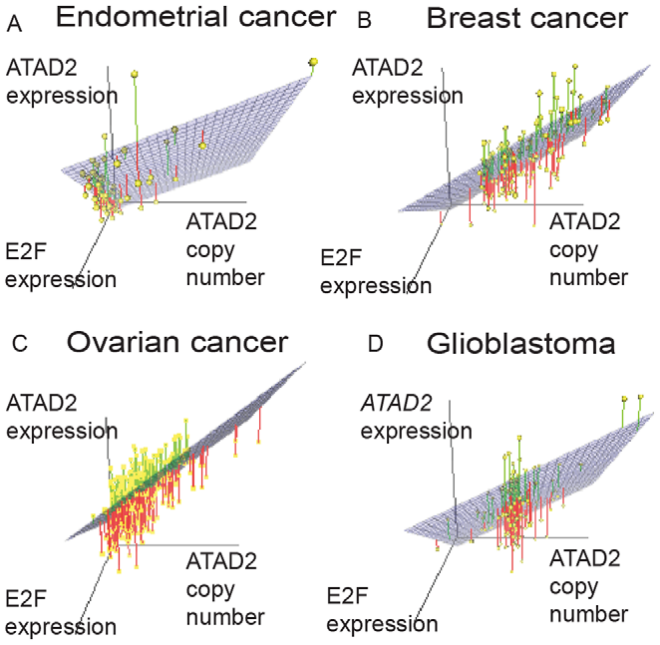
3-D-plots showing *ATAD2* expression and -copy-number and E2F1 expression. (a) Endometrial cancer, (b) breast cancer, (c) ovarian cancer, and (d) glioblastoma. Yellow dots represent the samples and the blue plate is the predicted 3-D fit. The green and red lines are the distance between the predicted fit and the actual observations for samples above and below the 3D-fit plate, respectively.

We found that *ATAD2* copy-number, *ESR1* expression and *E2F1* expression explained 77% of the variation in *ATAD2* expression in endometrial cancer, and each of the predictor variables remained significantly associated with *ATAD2* expression in the adjusted model (Figure 3a and Table S7). We also found that *ATAD2* copy-number and *E2F* expression independently predicted *ATAD2* expression in breast cancer, ovarian cancer and glioblastoma (Figure 3b–d and Table S7). *ESR1*, which was less strongly associated with *ATAD2* expression, was significant in the adjusted model only in endometrial cancer (p = 0.016) and glioblastoma (p<0.001), not in ovarian or breast cancer. These data suggest that the copy-number of *ATAD2* is an important determinant of *ATAD2* expression even in the context of other cellular regulatory mechanisms.

### Dependency on *MYC* Predicts Dependency on *ATAD2* and Response to HDAC Inhibitors in Endometrial- and Breast Cancer Cells

**Figure 4:**
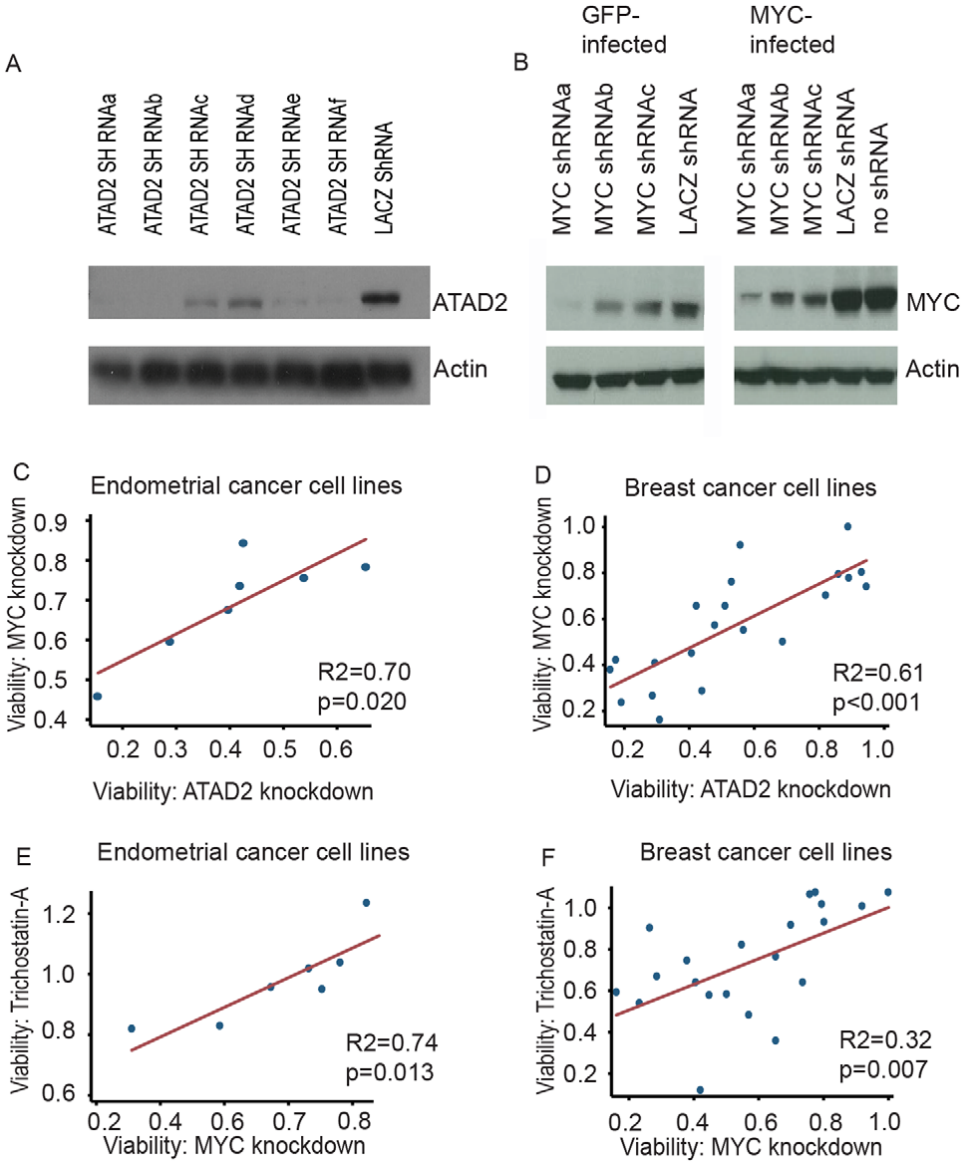
Correlation between effects of *ATAD2* and MYC knockdown. Western blots for (a) ATAD2 and (b) MYC indicate extent of knockdown with six shRNAs against *ATAD2* and three shRNAs against MYC, respectively. ATAD2 experiments were performed in KLE cells and MYC experiments were performed in TE9 cells infected with GFP control and MYC vectors. Subsequent experiments used *ATAD2* shRNAs a and e, and MYC shRNAs a and b. Reductions in cell viability among seven endometrial cancer cell lines (c) and 21 breast cancer cell lines (d) were highly correlated after knockdown of ATAD2 or MYC and after knockdown of MYC and treatment with the HDAC inhibitor Trichostatin-A (1.25 µM) (e–f).

The results above led us to hypothesize that *ATAD2* expression promotes MYC signaling and that endometrial cancer cells that are dependent upon *MYC* would also be dependent upon *ATAD2*. We measured the effect on viability of shRNA knockdowns of *ATAD2* and *MYC* in seven endometrial cancer cell lines. We used two shRNAs against each gene, selecting those that exhibited the greatest reduction of protein expression among six and three shRNAs screened against *ATAD2* and *MYC* respectively (Figure 4a, b).

Knockdown of either *ATAD2* or *MYC* resulted in highly correlated decreases in viability across the seven cell endometrial cancer lines (R2 = 0.70, p = 0.020; Figure 4c). In two cases, we observed over 75% reductions in viability. We found no association between expression of *ATAD2* or *MYC* or 8q24 copy number and sensitivity to *ATAD2* or *MYC* knockdown.

These results suggested that MYC-dependent cancers of other types might also be dependent on ATAD2. No decrease in proliferation had previously been seen with *ATAD2* knockdown in TIG3-T or U2OS cells [9]. When we tested a larger panel of 21 breast cancer lines, however, we confirmed the strong correlation between decrease in viability after knockdown of ATAD2 or MYC (R2 = 0.61, p<0.001; Figure 4d).

The association between dependency on *MYC* and *ATAD2* suggests ATAD2 as a therapeutic target in *MYC*-dependent cancers. Whereas *MYC* has long been a known oncogene, clinical approaches to block MYC signaling have not yet been successful. Histone deacetylase (HDAC) inhibitors, however, have been shown to indirectly inhibit bromodomain-containing proteins such as ATAD2 [24].

We used the Connectivity Map [25] to identify compounds whose signatures anticorrelated with the MYC signaling signature. Among the 1309 small molecules represented by the Connectivity Map, the signature of the HDAC inhibitor Trichostatin-A was most anticorrelated with the MYC signaling signature. (p-value<0.00001; Table S8). We also generated a signature of aggressive disease from the primary investigation series, using the 50 most over- and under-expressed genes in patients with metastatic disease compared to patients without metastatic disease. We found the Trichostatin-A signature was also the most anticorrelated with this signature of aggressive disease, tied with signatures of four other molecules (p<0.00001; Table S8).

To functionally confirm the relation between Trichostatin-A and MYC dependency, we tested all endometrial cancer and breast cancer cell lines for growth inhibition by Trichostatin-A and compared the results to *MYC* knockdown. Trichostatin-A inhibited growth in the same cell lines which were dependent on *MYC* both in the endometrial (R2 = 0.74, p = 0.013; Figure 4e), and in the breast cancer cell lines (R2 = 0.31, p = 0.007; Figure 4f) but the overall efficacy of Trichostatin-A at reducing cell viability was lower among the doses we tested (0.04–10 µM) (Table S9) than were the effects of *MYC* or *ATAD2* knockdown.

## Discussion

Our data suggest that *ATAD2* overexpression in human endometrial cancers is a consequence of 8q24 amplification and associated with MYC pathway activation. We also find that ATAD2 overexpression is associated with E2F activation and poor prognosis. Analyses of TCGA data suggest similar relationships between *ATAD2*, 8q24 amplification, and MYC pathway activation in glioblastoma, breast, and ovarian cancers. We also find that endometrial and breast cancer cell lines that are dependent upon MYC expression also depend upon expression of ATAD2.

High expression of *ATAD2* has previously been found to be associated with an unfavorable prognosis in breast, lung, and prostate cancers and it has been suggested that ATAD2 contributes to the development of aggressive cancer through linking of the *E2F* and *MYC* pathways [9], [10], [11]. We demonstrate an association between high *ATAD2* expression and negative outcome in endometrial cancer, using clinically well-characterized test and validation datasets. We also find that progression from primary to metastatic endometrial cancer is associated with a further increase of MYC signaling and *ATAD2* expression.

Ciro et al [9] previously showed that ATAD2 interacts with MYC in breast cancer cell lines and is overexpressed in 8q24 amplified breast cancers. Our results indicate that, in endometrial cancers, expression of *ATAD2* is more highly correlated with 8q24 amplification than is expression of its neighbors (including MYC), and that ATAD2 amplification and overexpression are strongly associated with multiple measures of MYC pathway activation in human tumors.

The finding of cooperative effects between *MYC* and coamplified genes on 8q24 is not entirely surprising. Indeed, the concept of oncogene cooperation was established through the study of positive interactions between *MYC* and other oncogenes such as *BCL2* [26]. Moreover, clustered genes are often functionally related [27]. The relevance of this phenomenon in cancer has been shown for the genes *MMP13*, *Birch2*, and *Birch3,* which are functionally related oncogenes contained on the same amplification in osteosarcoma [28], and for *BIRC2* and *YAP1*, cooperating oncogenes in an amplification in hepatocellular carcinomas [8].

Such a mechanistic association between *ATAD2* and *MYC*, and the finding that *MYC*-dependent cells are sensitive to *ATAD2* knockdown, suggest ATAD2 as a therapeutic target in MYC-dependent cancers. Although *MYC* has long been known as an oncogene [14] and is a promising drug target, it has not been successfully targeted therapeutically. Small molecule inhibitors have, however, been generated against other bromodomain-containing proteins [29]. Indeed, inhibition of the bromodomain-containing protein BRD4 has recently been suggested as an alternative approach to targeting MYC [30]. HDAC inhibitors also indirectly inhibit bromodomain-containing proteins by inducing histone hyperacetylation, thus probably diverting the specific bromodomain proteins from their targets [24]. This may account for some of the effectiveness of HDAC inhibitors as cancer therapeutics [30], and we found cell lines that were sensitive to knockdown of *MYC* or *ATAD2* were also sensitive to the HDAC inhibitor Trichostatin-A. However, the reduction in viability after application of Trichostatin-A was smaller than the reduction in viability after *MYC* or *ATAD2* knockdown. It is possible that a more direct inhibitor of ATAD2 would be more effective in these cells.

Major obstacles to treatment of patients with endometrial cancer include a lack of targeted therapeutics and of prognostic indicators. Indeed, endometrial cancer remains understudied relative to other cancer types. We find that *ATAD2* amplification and expression is a prognostic marker in endometrial cancer and our findings suggest that development of specific ATAD2 inhibitors is a promising approach to treatment of endometrial and other MYC driven cancers.

## Supporting Information

Figure S1 a) Five additional MYC activation genes sets obtained from the literature and their relative expression in 8q24 amplified versus unamplified samples. b) The correlation between MYC signaling strength and *MYC*- (left) and *ATAD2*- (right) gene expression in the primary investigation series and in the internal and external validation series. c) Estrogen receptor negative (ER−) tumors with *ATAD2* expression in the top quartile were associated with a high risk of disease-specific death; the risk was much lower among estrogen receptor positive (ER+) tumors with *ATAD2* expression in the bottom quartile. d-g) Results from the qPCR validation series. The copy-number of *MYC* and *ATAD2* genes are highly correlated in endometrial cancer (d), glioblastoma (e), ovarian cancer (f) and breast cancer (g).
(EPS)

Table S1 Details about the shRNA used in the study.
(DOCX)

Table S2 Names, origins and culture conditions for the cell lines used.
(DOCX)

Table S3 Patient characteristics and histopathological variables for the endometrial carcinoma series studied.
(DOCX)

Table S4 Genes in the *MYC* signaling signature.
(DOCX)

Table S5 Histopathological variables according to amplification of the 8q24 locus.
(DOCX)

Table S6 Gene expression of ATAD2 and MYC according to histopathological variables.
(DOCX)

Table S7 Prediction of ATAD2 gene expression by ATAD2 copy number, ESR1 gene expression and E2F1 gene expression.
(DOCX)

Table S8 Compounds with gene signatures anticorrelated to metastatic disease or the MYC signaling signature.
(DOCX)

Table S9 The associations between the sensitivity of 7 endometrial cancer cell lines to MYC knockdown and Tricostatin-A at different concentrations.
(DOCX)
