# Supplementary material for: Integrated Genomic Analysis of the 8q24 Amplification in Endometrial Cancers Identifies ATAD2 as Essential to MYC-Dependent Cancers
Source: PLoS One. 2013 Feb 5;8(2):e54873. doi: 10.1371/journal.pone.0054873 (PMC3564856; doi:10.1371/journal.pone.0054873)
Supplement: Table S1 — Details about the shRNA used in the study. (DOCX) [file pone.0054873.s002.docx]

S1: Details about the shRNA used in the study

| shRNA | TRC Identifier | NM number | Sequence (5’-3’) |
| --- | --- | --- | --- |
| ATAD2 shRNA a | TRCN0000158789 | NM_014109.2-4327s1c1 | GCCTAATTGATGTAGTATGAA |
| ATAD2 shRNA b | TRCN0000163392 | NM_014109.2-1324s1c1 | GCCGATGTTGATCCAATGCAA |
| ATAD2 shRNA c | TRCN0000158771 | NM_014109.2-4433s1c1 | GTCATGTGTAAGATAACTGAT |
| ATAD2 shRNA d | TRCN0000162701 | NM_014109.2-3109s1c1 | CGAGTGTTTACTAAGCCTGTT |
| ATAD2 shRNA e | TRCN0000161812 | NM_014109.2-4753s1c1 | CCAGAGTGCAAGTCATGATTT |
| ATAD2 shRNA f | TRCN0000159158 | NM_014109.2-563s1c1 | GTAGGATTAGAAGTCGTTATA |
| MYC shRNA a | TRCN0000039642 | NM_002467.2-1377s1c1 | CCTGAGACAGATCAGCAACAA |
| MYC shRNA b | TRCN0000039639 | NM_002467.2-1552s1c1 | CCCAAGGTAGTTATCCTTAAA |
| MYC shRNA c | TRCN0000039640 | NM_002467.2-1657s1c1 | CAGTTGAAACACAAACTTGAA |
