# Supplementary material for: Integrated Genomic Analysis of the 8q24 Amplification in Endometrial Cancers Identifies ATAD2 as Essential to MYC-Dependent Cancers
Source: PLoS One. 2013 Feb 5;8(2):e54873. doi: 10.1371/journal.pone.0054873 (PMC3564856; doi:10.1371/journal.pone.0054873)
Supplement: Table S2 — Names, origins and culture conditions for the cell lines used. (DOCX) [file pone.0054873.s003.docx]

| S2: Names, origins and culture conditions for the cell lines used | | | | | | |
| --- | --- | --- | --- | --- | --- | --- |
|  |  |  |  |  |  |  |
| **Name** | **Origin** | **Culture condition** | |  |  |  |
|  |  |  |  |  |  |  |
| ***Endometrial cancer cell lines*** | | | |  |  |  |
| AN3CA | ATCC | EMEM + 10%FBS | |  |  |  |
| Ishikawa | ECACC | MEM + 1% Non Essential Amino Acids (NEAA) + 15% FBS | | | |  |
| EFE-184 | ECACC | RPMI 1640 + 10% FBS | |  |  |  |
| HEC1A | ATCC | McCoy5a + 15% FBS | |  |  |  |
| KLE | ATCC | DMEM-E12 + 10% FBS | |  |  |  |
| MFE-296 | ECACC | 40% RPMI 1640+ 40% MEM with earles salt + 1x insulin-transferrin-sodium selenite + 20% FBS | | | | |
| HEC1B | ATCC | EMEM + 10 FBS | |  |  |  |
|  |  |  |  |  |  |  |
| ***Breast cancer cell lines*** | | |  |  |  |  |
| EVSA-T | DSMZ | RPMI 1640 + 10% FBS | |  |  |  |
| BT-549 | ATCC | RPMI 1640 + 0.23 units/ml human Insulin + 10% FBS | | | |  |
| CAL-85-1 | DSMZ | DMEM + 10% FBS | |  |  |  |
| HMC-1-8 | HSRRB | RPMI 1640 + 10% FBS | |  |  |  |
| Hs 578T | ATCC | DMEM + 0.01 mg bovine insulin + 10% FBS | | |  |  |
| MDA-MB-231 | ATCC | RPMI-1640 + 10% FBS | |  |  |  |
| DMS 273 | Sigma | Waymouth's MB + 10% FBS | | |  |  |
| MCF7 | ATCC | EMEM + 0.01 mg/ml bovine insulin + 10% FBS | | | |  |
| HCC38 | ATCC | RPMI 1640 + 10% FBS | |  |  |  |
| MDA-MB-436 | ATCC | RPMI 1640 + 10ug/ml bovine insulin + 16ug/ml glutathione + 10% FBS | | | | |
| MDA-MB-453 | ATCC | Leibovitz's L-15 + 10% FBS | | |  |  |
| BT-20 | ATCC | EMEM + 10% FBS | |  |  |  |
| AU565 | ATCC | RPMI 1640 + 10% FBS | |  |  |  |
| HCC1143 | ATCC | RPMI 1640 + 10% FBS | |  |  |  |
| HCC1954 | ATCC | RPMI 1640 + 10% FBS | |  |  |  |
| MDA-MB-175-VII | ATCC | RPMI 1640 + 10% FBS | |  |  |  |
| BT-474 | ATCC | Hybricare medium + 1.5 g/L Sodium Bicarbonate + 10% FBS | | | | |
| EFM-192A | DSMZ | RPMI 1640 + 20% FBS | |  |  |  |
| EFM-19 | DSMZ | RPMI-1640 ATCC catalog # 30-2001 | | |  |  |
| T-47D | ATCC | RPMI 1640 + 0.2 Units/ml bovine insulin + 10% FBS | | | |  |
| MDA-MB-468 | ATCC | DMEM + 10% FBS | |  |  |  |
|  |  |  |  |  |  |  |
| All culture media were also added: 10mM Glutamine and Penicillin-Streptomycin (Sigma) | | | | | | |
