# Supplementary material for: Integrated Genomic Analysis of the 8q24 Amplification in Endometrial Cancers Identifies ATAD2 as Essential to MYC-Dependent Cancers
Source: PLoS One. 2013 Feb 5;8(2):e54873. doi: 10.1371/journal.pone.0054873 (PMC3564856; doi:10.1371/journal.pone.0054873)
Supplement: Table S3 — Patient characteristics and histopathological variables for the endometrial carcinoma series studied. (DOCX) [file pone.0054873.s004.docx]

S3: Patient characteristics and histopathological variables for the endometrial carcinoma series studied.

|  |  |  | **Primary Investigation Series N=82** | |  | **Internal Validation Series N=59** | |  | **qPCR series N=162** | |  | **FISH validation series N=399** | |
| --- | --- | --- | --- | --- | --- | --- | --- | --- | --- | --- | --- | --- | --- |
|  |  |  | **N** | **%** |  | **N** | **%** |  | **N** | **%** |  | **N** | **%** |
| Follow-up time > 23 months* | | | | |  |  |  |  |  |  |  |  |  |
|  | Yes |  | 49 | 100% |  | 1 | 4% |  | 77 | 69% |  | 251 | 89% |
|  | No |  | 0 | 0% |  | 27 | 96% |  | 35 | 31% |  | 31 | 11% |
| Tumor sample | | |  |  |  |  |  |  |  |  |  |  |  |
|  | Primary tumor | | 82 | 100% |  | 40 | 68% |  | 162 | 100% |  | 399 | 100% |
|  | Metastatic lesion | | 0 | 0% |  | 19 | 32% |  | 0 | 0% |  | 0 | 0% |
| FIGO | |  |  |  |  |  |  |  |  |  |  |  |  |
|  | Stage I/II | | 69 | 84% |  | 31 | 78% |  | 129 | 80% |  | 334 | 84% |
|  | Stage III/IV | | 13 | 16% |  | 9 | 23% |  | 33 | 20% |  | 65 | 16% |
| Histology | | |  |  |  |  |  |  |  |  |  |  |  |
|  | Endometrioid | | 72 | 88% |  | 30 | 75% |  | 132 | 81% |  | 335 | 84% |
|  | Non-endometrioid | | 10 | 12% |  | 10 | 25% |  | 30 | 19% |  | 64 | 16% |
| Grade | | |  |  |  |  |  |  |  |  |  |  |  |
|  | Low/medium | | 58 | 73% |  | 15 | 37% |  | 106 | 66% |  | 275 | 69% |
|  | High |  | 22 | 28% |  | 25 | 63% |  | 55 | 34% |  | 122 | 30% |
| Estrogen receptor | | |  |  |  |  |  |  |  |  |  |  |  |
|  | Positive | | 61 | 73% |  | na |  |  | 112 | 82% |  | 166 | 77% |
|  | Negative | | 21 | 26% |  | na |  |  | 25 | 18% |  | 49 | 23% |
|  |  |  |  |  |  |  |  |  |  |  |  |  |  |
| *Among living patients with non-recurrent disease. We used 23 months representing mean time to recurrence as cut-off. | | | | | | | | | | | | | |
| Abbreviations: na= not available. | | | | | | | | | | | | |  |
|  | | | | | | |  |  |  |  |  |  |  |
