# Supplementary material for: Integrated Genomic Analysis of the 8q24 Amplification in Endometrial Cancers Identifies ATAD2 as Essential to MYC-Dependent Cancers
Source: PLoS One. 2013 Feb 5;8(2):e54873. doi: 10.1371/journal.pone.0054873 (PMC3564856; doi:10.1371/journal.pone.0054873)
Supplement: Table S4 — Genes in the MYC signaling signature. (DOCX) [file pone.0054873.s005.docx]

S4: Genes in the *MYC* signaling signature

| Gene symbol | | Gene Symbol | | Gene Symbol |
| --- | --- | --- | --- | --- |
| CLPB |  | SNRPB |  | TRAF2 |
| EFTUD2 |  | SNRPD2 |  | TXN |
| FEZ2 |  | SNRPD3 |  | TXNL4A |
| HOMER1 |  | SREBF1 |  | TXNRD1 |
| LOC10431 |  | SRM |  | TYMS |
| RRP9 |  | SRPK1 |  | UAP1 |
| RUVBL2 |  | STAT5B |  | UBE2C |
| SEPHS2 |  | SUMO2 |  | UCHL1 |
| SERPINE1 |  | SURF6 |  | UCK2 |
| SFRS1 |  | SYNGR1 |  | UCN2 |
| SFRS2 |  | TARBP1 |  | UMPS |
| SFRS7 |  | TBL3 |  | UNC119 |
| SFXN1 |  | TCF3 |  | UXT |
| SHMT1 |  | TDP1 |  | VARS |
| SLC16A1 |  | TERT |  | VDAC1 |
| SLC20A1 |  | TFDP1 |  | VRK1 |
| SLC25A3 |  | TFRC |  | WDR12 |
| SLC25A4 |  | THOP1 |  | WDR3 |
| SLC2A1 |  | THRA |  | YWHAE |
| SLC6A1 |  | TK1 |  | ZNF239 |
| SLC7A5 |  | TLE4 |  | ZNF330 |
| SMN1 |  | TOP1 |  | ZNF532 |
| SNRNP70 |  | TP53 |  |  |
