# Supplementary material for: Integrated Genomic Analysis of the 8q24 Amplification in Endometrial Cancers Identifies ATAD2 as Essential to MYC-Dependent Cancers
Source: PLoS One. 2013 Feb 5;8(2):e54873. doi: 10.1371/journal.pone.0054873 (PMC3564856; doi:10.1371/journal.pone.0054873)
Supplement: Table S5 — Histopathological variables according to amplification of the 8q24 locus. (DOCX) [file pone.0054873.s006.docx]

S5: Histopathological variables according to amplification of the 8q24 locus.

|  |  | **Primary Investigation Series** | | | | | | | | | **FISH Validation Series** | | | | | | | | | | |
| --- | --- | --- | --- | --- | --- | --- | --- | --- | --- | --- | --- | --- | --- | --- | --- | --- | --- | --- | --- | --- | --- |
|  |  | **No 8q24 amplification** | | | | **8q24 amplification** | | | |  |  | | **No 8q24 amplification** | | | | **8q24 amplification** | | | |  |
| **Characteristic N** | | | | **%** | **N** | | **%** | | **p-value** | | | **N** | | | **%** | **N** | | | **%** | **p-value** | |
| FIGO | |  |  | | |  | |  | | 0.16 |  | |  |  | | |  |  | | | 0.003 |
|  | Stage I/II | 48 | 89 | | | 12 | | 75 | |  |  | | 322 | 85 | | | 12 | 60 | | |  |
|  | Stage III/IV | 6 | 11 | | | 4 | | 25 | |  |  | | 57 | 15 | | | 8 | 40 | | |  |
| Histology | |  |  | | |  | |  | | <0.001 |  | |  |  | | |  |  | | | <0.001 |
|  | Endometrioid | 53 | 98 | | | 10 | | 63 | |  |  | | 324 | 85 | | | 11 | 55 | | |  |
|  | Non-endometrioid | 1 | 2 | | | 6 | | 38 | |  |  | | 55 | 15 | | | 9 | 45 | | |  |
| Grade | |  |  | | |  | |  | | <0.001 |  | |  |  | | |  |  | | | <0.001 |
|  | Low/medium | 48 | 92 | | | 4 | | 25 | |  |  | | 272 | 72 | | | 3 | 15 | | |  |
|  | High | 4 | 8 | | | 12 | | 75 | |  |  | | 105 | 28 | | | 17 | 85 | | |  |
| Estrogen Receptor | |  |  | | |  | |  | | 0.06 |  | |  |  | | |  |  | | | 0.29 |
|  | Positive | 43 | 80 | | | 9 | | 56 | |  |  | | 149 | 81 | | | 6 | 67 | | |  |
|  | Negative | 11 | 20 | | | 7 | | 44 | |  |  | | 35 | 19 | | | 3 | 33 | | |  |
