# Supplementary material for: Integrated Genomic Analysis of the 8q24 Amplification in Endometrial Cancers Identifies ATAD2 as Essential to MYC-Dependent Cancers
Source: PLoS One. 2013 Feb 5;8(2):e54873. doi: 10.1371/journal.pone.0054873 (PMC3564856; doi:10.1371/journal.pone.0054873)
Supplement: Table S6 — Gene expression of ATAD2 and MYC according to histopathological variables. (DOCX) [file pone.0054873.s007.docx]

S6: Gene expression of ATAD2 and MYC according to histopathological variables.

|  |  |  |  |  |  |  |  |  |  |  |  |  |  |  |  |  |  |  |
| --- | --- | --- | --- | --- | --- | --- | --- | --- | --- | --- | --- | --- | --- | --- | --- | --- | --- | --- |
|  |  | **Primary investigation series; n=82** | | | | | | | |  | **qPCR validation series; n=162** | | | | | | | |
|  |  |  | *ATAD2* gene expression | | |  | *MYC* gene expression | | |  |  | *ATAD2* gene expression | | |  | *MYC* gene expression | | |
| Characteristic | | N | Mean | SD | p-value |  | Mean | SD | p-value |  | N | Mean | SD | p-value |  | Mean | SD | p-value |
| FIGO | |  |  | 0.2 | |  |  |  | 0.3 |  |  |  |  | 0.032 |  |  |  | 0.7 |
|  | Stage I/II | 68 | 7.32 | 0.57 |  |  | 7.87 | 0.29 |  |  | 129 | 0.54 | 0.39 |  |  | 0.65 | 0.64 |  |
|  | Stage III/IV | 14 | 7.52 | 0.52 |  |  | 7.98 | 0.5 |  |  | 33 | 0.74 | 0.68 |  |  | 0.7 | 0.8 |  |
| Histology | |  |  |  | 0.03 |  |  |  | 0.03 |  |  |  |  | <0.001 |  |  |  | 0.08 |
|  | Endometrioid | 72 | 7.3 | 0.57 |  |  | 7.86 | 0.33 |  |  | 132 | 0.51 | 0.35 |  |  | 0.61 | 0.63 |  |
|  | Non-endometrioid | 10 | 7.72 | 0.33 |  |  | 8.11 | 0.28 |  |  | 30 | 0.89 | 0.74 |  |  | 0.85 | 0.81 |  |
| Grade | |  |  | <0.001 | |  |  |  | <0.001 |  |  |  |  | 0.005 |  |  |  | 0.02 |
|  | Low/medium | 58 | 7.15 | 0.42 |  |  | 7.82 | 0.29 |  |  | 106 | 0.51 | 0.38 |  |  | 0.56 | 0.43 |  |
|  | High | 22 | 7.9 | 0.54 |  |  | 8.09 | 0.37 |  |  | 55 | 0.73 | 0.59 |  |  | 0.83 | 0.97 |  |
| Estrogen receptor | |  |  |  | 0.02 |  |  |  | 0.06 |  |  |  |  | 0.02 |  |  |  | 0.3 |
|  | Positive | 57 | 7.26 | 0.52 |  |  | 7.86 | 0.3 |  |  | 112 | 0.54 | 0.39 |  |  | 0.69 | 0.72 |  |
|  | Negative | 19 | 7.61 | 0.59 |  |  | 8.02 | 0.33 |  |  | 25 | 0.79 | 0.71 |  |  | 0.53 | 0.43 |  |
