# Supplementary material for: Integrated Genomic Analysis of the 8q24 Amplification in Endometrial Cancers Identifies ATAD2 as Essential to MYC-Dependent Cancers
Source: PLoS One. 2013 Feb 5;8(2):e54873. doi: 10.1371/journal.pone.0054873 (PMC3564856; doi:10.1371/journal.pone.0054873)
Supplement: Table S7 — Prediction of ATAD2 gene expression by ATAD2 copy number, ESR1 gene expression and E2F1 gene expression. (DOCX) [file pone.0054873.s008.docx]

S7: Prediction of ATAD2 gene expression by ATAD2 copy number, ESR1 gene expression and E2F1 gene expression

|  |  | **Unadjusted model** | |  | **Adjusted model*** | |
| --- | --- | --- | --- | --- | --- | --- |
|  |  | R2 | p-value |  | R2 | p-value |
| **Endometrial cancer** | |  |  |  | 0.76 |  |
|  | *ATAD2* copy number | 0.36 | <0.001 |  |  | <0.001 |
|  | *ESR1* gene expression | 0.1 | 0.005 |  |  | 0.018 |
|  | *E2F1* gene expression | 0.63 | <0.001 |  |  | <0.001 |
|  |  |  |  |  |  |  |
| **Breast cancer** | |  |  |  | 0.65 |  |
|  | *ATAD2* copy number | 0.47 | <0.001 |  |  | <0.001 |
|  | *ESR1* gene expression | 0.07 | <0.001 |  |  | 0.15 |
|  | *E2F1* gene expression | 0.43 | 0.002 |  |  | <0.001 |
|  |  |  |  |  |  |  |
| **Ovarian cancer** | |  |  |  | 0.43 |  |
|  | *ATAD2* copy number | 0.36 | <0.001 |  |  | <0.001 |
|  | *ESR1* gene expression | 0.01 | 0.13 |  |  | 0.061 |
|  | *E2F1* gene expression | 0.1 | <0.001 |  |  | <0.001 |
|  |  |  |  |  |  |  |
| **Glioblastoma** | |  |  |  | 0.52 |  |
|  | *ATAD2* copy number | 0.11 | <0.001 |  |  | <0.001 |
|  | *ESR1* gene expression | 0.16 | <0.001 |  |  | <0.001 |
|  | *E2F1* gene expression | 0.39 | <0.001 |  |  | <0.001 |
|  |  |  |  |  |  |  |
| * Adjusted for *ATAD2* copy number, *ESR1* gene expression and *E2F1* gene expression | | | | | |  |
