# Supplementary material for: Integrated Genomic Analysis of the 8q24 Amplification in Endometrial Cancers Identifies ATAD2 as Essential to MYC-Dependent Cancers
Source: PLoS One. 2013 Feb 5;8(2):e54873. doi: 10.1371/journal.pone.0054873 (PMC3564856; doi:10.1371/journal.pone.0054873)
Supplement: Table S8 — Compounds with gene signatures anticorrelated to metastatic disease or the MYC signaling signature. (DOCX) [file pone.0054873.s009.docx]

S8: Compounds with gene signatures anticorrelated to metastatic disease or the MYC signaling signature

| **Rank** | **Name of compound** | **Known function** | **N*** | | **p-value†** | |
| --- | --- | --- | --- | --- | --- | --- |
|  |  |  |  |  |  |  |
|  |  |  |  | |  | |
| Anti-correlated to the MYC signaling signature | | |  | |  | |
|  |  |  |  | |  | |
| 1 | trichostatin A | HDAC inhibitor | 182 | | <0.00001 | |
| 1 | LY-294002 | PI3-Kinase inhibitor | 61 | | <0.00001 | |
| 1 | sirolimus | mTOR inhibitor | 44 | | <0.00001 | |
| 1 | tanespimycin | heat shock protein 90 inhibitor | 62 | | <0.00001 | |
| 5 | trifluoperazine | antipsychotic drug | 16 | | 0.0006 | |
| 6 | metyrapone | steroid 11β-hydroxylase inhibitor | 4 | | 0.0007 | |
| 7 | latamoxef | oxacephem antibiotic | 3 | | 0.0001 | |
| 8 | 3-acetylcoumarin |  | 5 | | 0.001 | |
| 9 | wortmannin | PI3-Kinase inhibitor | 18 | | 0.001 | |
| 10 | vorinostat | HDAC inhibitor | 12 | | 0.003 | |
|  |  |  |  | |  | |
| Anti-correlated to a gene signature defined by genes differentially regulated in patients with metastatic disease compared with patients without metastatic disease | | | | | |  |
|  |  |  |  |  |  |  |
|  |  |  |  | |  | |
| 1 | puromycin | Protein synthesis inhibitor | 4 | | <0.00001 | |
| 1 | cicloheximide | Protein synthesis inhibitor | 4 | | <0.00001 | |
| 1 | trichostatin A | HDAC inhibitor | 182 | | <0.00001 | |
| 1 | sirolimus | mTOR inhibitor | 44 | | <0.00001 | |
| 1 | LY-294002 | PI3-Kinase inhibitor | 61 | | <0.00001 | |
| 6 | wortmannin | PI3-Kinase inhibitor | 18 | | 0.00002 | |
| 7 | thioridazine | Antipsychotic drug | 20 | | 0.00006 | |
| 8 | cephaeline | alkaloid chemical | 5 | | 0.0003 | |
| 9 | vorinostat | HDAC inhibitor | 12 | | 0.0003 | |
| 10 | trifluoperazine | Antipsychotic drug | 16 | | 0.0004 | |
|  |  |  |  | |  | |
|  |  |  |  | |  | |
| * N = number of instances in which the compounds were tested in the Connectivity map | | | |  | |  |
| †The p-value for each small molecule represents the distribution of these scores | | |  | |  | |
| compared with the distribution of scores among all small molecules, using a | | |  | |  | |
| permutation test as described by Lamb et al (2) | | |  | |  | |
