# Supplementary material for: Integrated Genomic Analysis of the 8q24 Amplification in Endometrial Cancers Identifies ATAD2 as Essential to MYC-Dependent Cancers
Source: PLoS One. 2013 Feb 5;8(2):e54873. doi: 10.1371/journal.pone.0054873 (PMC3564856; doi:10.1371/journal.pone.0054873)
Supplement: Table S9 — The associations between the sensitivity of 7 endometrial cancer cell lines to MYC knockdown and Tricostatin-A at different concentrations. (DOCX) [file pone.0054873.s010.docx]

S9: The associations between the sensitivity of 7 endometrial cancer cell lines to MYC knockdown and Tricostatin-A at different concentrations.

|  | **Association to viability after MYC knockdown** | |
| --- | --- | --- |
|  |  |  |
| **Consentration of Tricostatin-A** | **R2** | **P-value** |
|  |  |  |
| 0.04 μM | 0.55 | 0.091 |
| 0.16 μM | 0.49 | 0.079 |
| 0.60 μM | 0.50 | 0.074 |
| 1.25 μM | 0.75 | 0.013 |
| 5.0 μM | 0.94 | <0.001 |
| 10 μM | 0.62 | 0.034 |
